# Implementation of a Participatory Ergonomics Intervention to Reduce Musculoskeletal and Stress-Related Mental Health Risks in Australian Retail Workers: Protocol for a Randomized Controlled Trial

**DOI:** 10.2196/84864

**Published:** 2026-01-29

**Authors:** Elise Condie, Victoria Weale, Katrina A Lambert, Jodi Oakman

**Affiliations:** 1 Centre for Ergonomics and Human Factors Department of Public Health La Trobe University Bundoora, Victoria Australia

**Keywords:** ergonomics, implementation science, musculoskeletal diseases, occupational stress, randomized controlled trial

## Abstract

**Background:**

Worker participation has been identified as important for managing the risks of work-related musculoskeletal disorders (WMSDs) and stress-related mental health problems (MHPs). Previously identified barriers include securing long-term management support to implement risk reduction measures. Few studies evaluate how a manager or decision maker’s readiness to act influences the outcomes of a participatory ergonomics program. The Stages of Change (SoC) framework has been suggested for tailoring ergonomics interventions to managers’ receptiveness in a workplace setting.

**Objective:**

The main aim is to evaluate the implementation of the “A Participatory Hazard Identification and Risk Management” (APHIRM) toolkit in the online order fulfillment department for a sample of stores in a large retail organization, compared to usual risk management practice.

**Methods:**

This study is a cluster quasi–randomized controlled trial, comparing implementation of the APHIRM toolkit with usual safety risk management practice. As is typical for workplaces, the intervention is facilitated by the organization’s safety team. We recruited 9 control and 9 intervention stores to the study through random selection of eligible stores. Quantitative data are collected at baseline and 12-month follow-up. Qualitative data to enable a process evaluation are collected over the duration of the study. Primary outcome measures are physical and psychosocial hazard severity scores. Secondary outcomes are self-rated pain and discomfort scores and action plan implementation measures. Managers’ progression through SoC is an additional outcome measure. The primary outcome measures (physical and psychosocial hazard severity ratings) will be analyzed by variance-weighted cluster-level ANCOVA. Ethics approval was granted by the La Trobe University Human Research Ethics Committee (HEC25088).

**Results:**

Funding was provided in June 2025. Recruitment and randomization concluded in early August 2025. The intervention, including data collection, commenced in late August 2025 and is expected to conclude in September 2026. A total of 332 participants have been recruited to the study. Response rates have averaged 46% across control and intervention groups. As of January 2026, no data analyses have been conducted. Primary findings are anticipated to be published in Spring 2028.

**Conclusions:**

This study evaluates the implementation of the APHIRM toolkit survey in a multisite, large retail organization in Australia and describes the use of toolkit resources. It evaluates managers’ SoC regarding WMSD and MHP prevention and how this may influence outcomes. Findings from this study should provide additional insight on how to implement the toolkit in large organizations to reduce WMSD and stress-related MHP risk and inform future development of the content of the APHIRM toolkit. This study is anticipated to further inform tailoring of interventions to managers’ and decision-makers’ SoC.

**Trial Registration:**

OSF Registries 10.17605/OSF.IO/82R9G; https://osf.io/82r9g

**International Registered Report Identifier (IRRID):**

DERR1-10.2196/84864

## Introduction

### Background

Work-related musculoskeletal disorders (WMSDs) are one of the most frequent and costly type of compensable injuries in Australia, representing 87% of serious claims for injury or disease during 2019-2020 [1]. “Body stressing” represented 50% of all serious claims lodged in the retail trade sector from 2000 to 2021 [2]. Previous research has indicated that employers and ergonomists remain predominantly focused on controlling the biomechanical hazards associated with WMSDs [3-6]. This is despite consensus that psychosocial hazard exposures also contribute to these injuries and that exposure to multiple hazards simultaneously can have an additive or interactive effect [7-9]. Workplace psychosocial hazard exposure can also lead to a stress response in workers, which can in turn lead to mental health problems (MHPs) [10-13].

Effective WMSD and MHP prevention requires employers to address both the physical and psychosocial sources of injury risk. This requires accounting for the demands of the entire job, rather than evaluating and modifying individual tasks [4,8]. Workforce participation in all steps of the risk management process has also been identified as important to reducing WMSD risk exposure [14-16]. Multifactorial interventions, that is, multiple simultaneous actions to address sources of risk, selected from multiple levels of the hierarchy of controls, have also been supported as effective in reducing WMSD risk [17-20]. Multifactorial interventions are also supported for prevention of MHPs [21-23]. However, employer practices continue to be misaligned with the evidence regarding effective WMSD and MHP prevention [5,6,24,25].

Previous research has identified that employers’ approaches to both physical and psychosocial hazard and risk management are typically individualistic rather than system based. These studies have found that implemented changes rarely address the source of injury risk and are therefore unlikely to be effective at preventing WMSDs and MHPs [23]. A review of available WMSD risk management tools [26] identified only 3 comprehensive tools (ie, included both physical and psychosocial risk factors), that also used participative methods to facilitate all stages of the risk management process.

One of the 3 tools identified in the review [26] was “A Participatory Hazard Identification and Risk Management” (APHIRM) toolkit. This is a set of procedures that guides employers and workers through the 5 stages of the risk management cycle to address the sources of WMSD and MHP risk specific to a particular job. These procedures are implemented with guidance from a facilitator who has been trained to implement the toolkit. The procedures focus on implementing changes that address the sources of risks of WMSDs and MHPs. Studies evaluating the toolkit have supported its effectiveness in reducing the severity of exposure to physical and psychosocial hazards that can contribute to WMSDs and MHPs [27-29].

Effective WMSD and MHP risk reduction relies on management support, participation, and commitment of resources to implement identified actions [5,14,30,31]. Aligning approaches with management and workforce readiness for change has been suggested to improve the outcomes resulting from these changes [19,32-34]. The Stages of Change model (SoC) [35] is one approach to adapting the delivery of interventions in the workplace to the recipient’s readiness to change [32,36]. This model classifies individuals into one of five SoC: (1) precontemplation (unaware or unconcerned about workplace hazards), (2) contemplation (considering change but not yet ready to act), (3) preparation (intent to change in the near future), (4) action (made changes in the previous 6 months), and (5) maintenance (made changes and are working to consolidate gains and avoid relapse).

An individual’s SoC is determined by their current knowledge, attitudes, and beliefs regarding the issue in focus. The key constructs thought to influence movement through the stages are decisional balance, that is, weighing the pros and cons of changing, and habit strength, that is, the ability to sustain a target behavior over time [37,38]. A scoping review [39] identified few intervention studies performed in a workplace setting that used the SoC model. Previous studies on WMSD prevention [36,40,41] have used the SoC model to evaluate whether advice and interventions tailored to the target’s SoC are more effective at addressing WMSD hazards in the workplace. In this earlier work, both the managers’ and employees’ SoC were evaluated simultaneously, using a short questionnaire. Those in earlier SoC made a greater number of successful ergonomics changes when advice was tailored to their SoC. This suggests that such an approach has promise for improving the effectiveness of interventions aimed at making ergonomics changes in the workplace. Less attention has been paid specifically to the relationship between managers’ or decision-makers’ SoC (as a proxy for readiness to make changes) and the success of ergonomics interventions implemented in their area of control [19,31,36].

There is an opportunity to implement the APHIRM toolkit in a large retail organization and assess its impact on WMSD and MHP hazard exposure. The process evaluation conducted in this study will inform more effective toolkit implementation in organizations of similar scale and/or risk profile, accounting for managers’ SoC. Implementation science methodologies have been recommended as an approach to a process evaluation [26,42,43], to improve robustness, while maintaining a “real-world” context. This study intends to build on available evidence relating to effective WMSD and MHP risk management obtained from previous workplace-based studies, particularly those which have implemented the APHIRM toolkit. There are few published studies that describe the implementation of the APHIRM toolkit in large workplaces [44,45].

### Study Objectives

To address these gaps, the aims of this research are to evaluate the impact of APHIRM toolkit implementation on hazard exposure levels for workers in the online shopping department (online department), conduct a process evaluation of the toolkit implementation in each participating work group, and evaluate the influence of SoC on the use of APHIRM toolkit resources.

To achieve these aims, this research protocol is designed to answer the following questions: does implementation of the APHIRM toolkit reduce hazard exposure levels for workers in the online department? When implementing the APHIRM toolkit, what are the key requirements to maximize its effectiveness in reducing hazard exposures for workers in the online department? Are APHIRM toolkit resources used differently when senior leaders have different SoC?

## Methods

### Ethical Considerations

Ethics approval was granted by the La Trobe University Human Research Ethics Committee in July 2025 (approval HEC25088). Survey participants were provided with consent information before commencing the relevant surveys. These included the SoC survey for managers and the APHIRM toolkit survey for online team members at the stores included in the study. Survey response data were anonymized as part of the collection process. Participants in the risk management teams (RMTs) of the intervention group were provided with a participant informed consent survey prior to taking part in the study, as were participants taking part in interviews for the process evaluation component of the study. RMT data were deidentified at the completion of data collection. Participants completed activities related to the study during paid work time but were not provided with additional compensation for their participation.

### Registration

The trial has been registered through the Open Science Framework (osf.io/82r9g). Registration was performed after completion of recruitment and following commencement of data collection, but before data were viewed by the research team. Data collection commenced prior to finalization of trial registration to ensure that key activities in the intervention did not take place over the busy Christmas and new year period. Attempting to do so would have likely threatened survey response rates and stores’ level of participation in the intervention overall. Although ideally trial registration would have been completed prior to commencement of data collection, this outcome reflects the challenges associated with conducting research in a live workplace, including maintaining project momentum and sustaining organizational stakeholder engagement and motivation.

### Trial Design

This is a cluster quasi–randomized controlled trial. Each cluster comprises the employees and managers of an online order fulfilment department (known hereafter as the “online department”) in an individual store in a large retail organization. Clusters are described as “work groups” for the purposes of this study. The ratio of intervention work groups to control work groups is 1:1.

The APHIRM toolkit is designed so that nonexperts (ie, individuals other than ergonomists) can implement WMSD and MHP risk reduction measures in their workplaces. On this basis, a facilitator from the organization’s health and safety team is assigned to guide toolkit implementation for each work group in the intervention arm. Assignment of facilitators to work groups is based on their geographic location, relative to the location of the store. Facilitators are overseen by the organization’s ergonomics team, who monitor adherence to toolkit process and procedure. The organization’s ergonomics team are, in turn, overseen by the lead author, who is also employed by the organization in a health and safety role. The lead author is also a member of the research team. The research team monitors the conduct of the study, including compliance with ethics requirements, and guides a robust approach to the study and the subsequent evaluation. This structure is intended to achieve a study design that most closely resembles the real-world conditions in which this intervention would typically occur. A lack of blinding at the cluster level could be considered a threat to the validity of the study; however, discourse of the research-to-practice gap in musculoskeletal disorder (MSD) prevention research [15,33,46] suggests a need for published data on interventions that most resemble a real-world implementation environment. This includes the conduct of research in real workplaces, on real workers, facilitated by those with the typical skill and expertise that would be encountered in a real-world setting [18]. This design reflects a previously identified challenge for workplace safety and ergonomics practitioners, to translate methodologically “correct” research into evidence-based prevention practice in the scope of their roles [23]. The risks of assessment bias are mitigated through review of data collection, analysis, and interpretation by the other members of the research team, who are not part of the intervention.

### Participants

Eligible clusters (“work groups”) are those stores with an online department, located within 1.5 hours’ drive of Sydney or Brisbane, Australia, which are the cities where the facilitators are based. Random sampling within eligible work groups will account for work groups with varying sales volumes and service offerings (eg, delivery to a customer’s vehicle parked outside the store, loading orders onto trucks for home delivery, and/or collection of orders by a third-party delivery driver through a service such as Uber). Managers of work groups are recruited to the study if they are the manager of the online department or store associated with the individual work group or are a manager responsible for the group of stores that the individual store or work group sits within.

Facilitators are allocated to work groups in a manner that replicates real-world conditions for toolkit implementation. These facilitators work in the organization’s health and safety team in the states where the intervention is conducted and possess a range of qualifications and experience, from a background in store management and operations through to degree-qualified health and safety professionals with varying years of experience.

### Recruitment

A total of 18 work groups (9 intervention and 9 control) will be recruited to the study. All work groups will be located within 1.5 hours’ travel time of Sydney or Brisbane, Australia. Settings range from inner urban to semirural locations. The number of work groups required for the study was determined based on the average number of employees in each work group and typical response rates to employee engagement surveys in stores, which are usually 20%-30%.

Stores are recruited to the study through consultation with the resource capacity management unit within the organization. This unit determines which stores are already engaged in other activities which would limit their ability to participate. This unit will then facilitate communication with the management of the groups of stores deemed eligible for participation. This management team then facilitates communication with the stores in their group that are selected for participation via the randomization process. Recruitment of stores continues until the required sample size is achieved.

Individual participants within a work group are eligible for recruitment to the study if they work some or all their work time in the online department, are a manager of an eligible online department or the store, or a manager overseeing an eligible store or “group” of 10-11 stores. These individual employees are involved in the study through completion of the APHIRM toolkit survey and feedback and consultation processes on identified sources of risk and action plans. Recruitment materials and advertising will be distributed through channels only accessible to those employees working in the online department. This is to prevent inadvertent recruitment of employees working in other departments in the store. These channels include the digital discussion board used to communicate with online employees and the posting of physical flyers on the noticeboards in the online room of the store. Informed consent statements and information on the study are provided to participants when they access the online portal used to administer the toolkit.

Recruitment of managers will be conducted through meeting invitations for briefing sessions aimed at communicating the study or providing updates about the study’s progress. Informed consent statements are provided to these managers at the commencement of the sessions.

RMT members for each work group are recruited through the same channels as individual participants in the study. Each work group is encouraged to recruit a minimum of 3 RMT members, which is intended to ensure that at least 2 RMT members are available to participate in each of the monthly facilitated sessions in store.

### Intervention

Clusters in the experimental group will implement the APHIRM toolkit processes and procedures with the guidance of a facilitator. The APHIRM toolkit guides work groups through the steps of the risk management cycle to address the sources of physical and psychosocial hazards that contribute to WMSDs and MHPs. Each step of toolkit implementation is delivered as a face-to-face session lasting up to 2 hours. Resources used to facilitate these sessions are freely available on the official website [47].

The first step of the process involves identifying members of the work group to act as representatives for their peers during toolkit implementation. This group is known as an RMT.

In the second step, the work group is invited to complete a survey that rates exposure to physical and psychosocial hazards, musculoskeletal pain or discomfort, and stress levels. The survey forms the hazard identification and risk assessment component of the risk management process. This survey is administered through an online portal, hosted as part of the APHIRM toolkit on its official website [47]. This enables the completion of surveys on any computer or mobile device. The survey is anonymous at the individual level, with responses aggregated to the work group (store) level.

In the third step, the results of the survey are provided to the work group, who then provide input on the work-related sources of risk relating to the identified hazards. Over the course of 10 monthly face-to-face sessions, the facilitator guides the RMT through the development and implementation of an action plan to address the sources of risk. It is anticipated that RMTs will identify a combination of actions that are specific to their individual store, as well as actions that are implemented in every store and thus require assistance from personnel above the individual store level to address.

The implementation of actions at the store level is expected to assist in differentiating intervention stores from control stores when measuring hazard severity at follow-up. It is also possible that the act of engaging RMTs from the intervention stores in the development of above-store actions will have an impact on hazard severity scores at follow-up, greater than the impact of the actual implementation of changes to the system of work. The impacts of these changes are expected to be felt by control and intervention stores equally if they are implemented before follow-up data collection. Many changes at the system of work level are unlikely to be implemented before follow-up, further allowing for differentiation between control and intervention groups. If significant changes are implemented in both control and intervention stores during the study, and it is determined that these changes may influence the results of the study, an additional survey will be conducted following the implementation of this change. This will be followed by the planned survey at the 12-month follow-up. This 12-month follow-up comprises the fourth step of the intervention, in which the survey is repeated approximately 12 months after the initial survey. These results are also communicated to the work group, along with an invitation to provide feedback on their perceived effectiveness of the action plan.

In parallel with implementation of the APHIRM toolkit, managers who are responsible for the online department for each intervention work group will be invited to participate in a questionnaire to evaluate their SoC. This will be conducted at commencement of the intervention, then every 3 months during the intervention, and on conclusion of the study, approximately 12 months following commencement.

Clusters in the control arm do not implement the APHIRM toolkit. These work groups only complete the APHIRM toolkit survey at commencement and conclusion of the study, and the managers associated with these work groups complete the SoC assessment every 3 months during the intervention period. These work groups otherwise receive the usual care and safety support from their management team, including the safety professionals who usually support the work group. This includes conducting a monthly safety meeting with the work group’s safety committee, performing safety inspections, and participating in other routine activities relating to managing workplace health and safety in the store. Work groups assigned to the control arm are not prevented from implementing changes to address sources of MSD or MHP risk.

**Figure 1:**
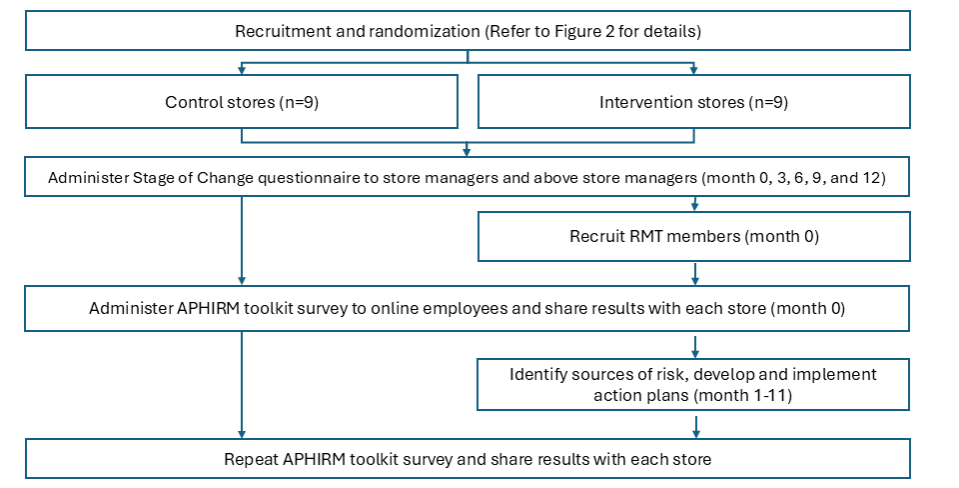
Structure and timing of intervention activities. APHIRM: A Participatory Hazard Identification and Risk Management; RMT: risk management team.

Figure 1 illustrates the structure and timing of the intervention and control work groups’ involvement in the study for the purposes of providing comparison data.

Facilitators from the organization’s health and safety team are trained in the administration of the intervention by a member of the research team. This includes training on the procedures to facilitate each of the sessions that comprise the intervention, as well as data collection and ethics compliance procedures. The training is delivered using the resources provided in the APHIRM toolkit. One member of the research team and one member of the health and safety team have completed the 1-day APHIRM training workshop offered by La Trobe University on establishing and delivering the APHIRM toolkit in workplaces.

Adherence to toolkit procedures is monitored by reviewing the outputs of the work group, which are recorded in the work group’s toolkit portal on APHIRM’s official website [47]. Facilitators use an online notebook to record observations from each session, including how clusters use the resources from the APHIRM toolkit, fidelity to the toolkit procedures, and other observations that are important to the process evaluation completed as part of the study.

If the data provided at the conclusion of a session indicate that procedures may not have been correctly followed, a member of the research team will complete a follow-up session with that facilitator and the work group in question to review and prepare the work group for the next session and step in the intervention.

### Outcomes

#### Quantitative Outcomes

Quantitative data are collected anonymously at the individual level and are therefore aggregated and analyzed at the work group level. Baseline and follow-up data are collected at the same time of the year (August/September), which assists in accounting for seasonal variation and avoids potential impacts on response rates by avoiding busy trading periods.

#### Primary Outcomes

Previous research has confirmed the validity of the toolkit survey [29] and generated evidence to support the toolkit’s effectiveness at reducing exposure to hazards that contribute to WMSDs and MHPs [27,28]. The primary outcome measures for this study are changes in the mean severity ratings for physical and psychosocial hazards, based on the relevant items from the APHIRM toolkit survey. These data are self-reported by members of the work group. Mean severity ratings for each group of hazard items are calculated at the cluster level (ie, each work group), by calculating the difference between baseline and follow-up means. All primary outcome measures will be assessed for mean change by cluster, which will be analyzed using ANCOVA, comparing outcomes for the 2 arms. Outcomes for each work group will also be compared to identify potential outliers. Data will be cleaned and examined for normal distribution prior to analysis. Differences in cluster sizes will be identified and accounted for using suitable analysis, which will be reported with the results.

#### Calculation of Severity Ratings for Physical Hazard Measures

A total of 12 survey items relate to physical (biomechanical) hazards that may influence MSD risk. Hazard measures include items related to force, repetition, and awkward and sustained postures, and vibration. Respondents rate their exposure to each hazard, in the last 6 months, on a scale from 1 (almost never) to 5 (almost always). A mean rating is calculated for all 12 survey items relating to physical hazard exposures.

#### Calculation of Severity Ratings for Psychosocial Hazard Measures

A mean rating for each cluster is calculated for all 44 survey items relating to psychosocial hazards. Examples of psychosocial hazard measures include items relating to control over the pace and content of work, role clarity, and reward and recognition.

#### Secondary Outcomes: Score for Self-Rated Pain and Discomfort

Using the measures in the APHIRM toolkit survey, participants rate the frequency and severity of their musculoskeletal pain and discomfort (in the last 6 months) in each of 5 body regions: neck/shoulders, arms, hands/fingers, middle/lower back, and hips/bottom/legs/feet. For example, “In the last six months, how often have you felt discomfort or pain in your neck or shoulders?” Response options range from 0 (never) to 4 (almost always). Any response equal to or greater than 1 (occasionally) then generates an additional question, for example “In the last six months, how bad was the discomfort or pain in your neck or shoulders?” Response options range from 1 (mild) to 3 (severe). A score out of 12 (4 × 3) is calculated for each body region and summed to produce a score out of 60 for each participant reporting any pain or discomfort. The change in mean scores between baseline and follow-up is used to evaluate outcomes.

#### Self-Rated Stress Score

A total of 12 of the APHIRM toolkit survey items are used to calculate a stress score. Eleven of these 12 items are from the Copenhagen Psychosocial Questionnaire [48]. The items relate to stress (“problems relaxing,” “been tense,” and “been irritable”), cognitive stress (“difficulty with thinking clearly,” “concentrating,” “making decisions,” and “remembering”), and burnout (“worn out,” “physically exhausted,” “tired,” and “emotionally exhausted”). The 12th survey item used to calculate a stress score is “had difficulty in falling or staying asleep.” This is from the General Well-Being Questionnaire [49], identified as a valid measure for stress in a workplace context, with more brevity than the equivalent items used in the Copenhagen Psychosocial Questionnaire [29]. Response options range from 0 (never) to 4 (almost always). A score out of 48 is calculated for each respondent by multiplying the ratings for each item (on a scale of 0 to 4). The change in mean scores between baseline and follow-up is used to evaluate outcomes.

#### Action Plan Implementation

Data on action plan implementation will be collected via inputs and updates to the action plan created and updated for each store and managed through the toolkit portal. Actions will be classified as “completed,” “in progress,” or “not started” through the review of the action plan during monthly sessions conducted with the facilitator and the work group. Actions will also be classified as “higher-order” actions, involving hazard elimination, substitution, or engineering actions, or “lower-order” actions, involving administrative actions such as process changes.

Actions will be classified as “store-level” or “above-store-level” actions by the facilitation team, partly to determine which actions require escalation above the store’s management to be addressed, and partly to evaluate how many store-level actions achieve “completed” status, compared with actions classified as above-store-level. This data will be validated by the research team through an independent review of action plan data collected by the APHIRM toolkit online portal. The count of each action and its classification in each of the above dimensions at the end of the 12-month study for each cluster will be reported.

#### Additional Outcome Measure

Managers’ SoC progression will be measured using the questionnaire included in Multimedia Appendix 1. The questionnaire will be administered every 3 months for the duration of the study via scheduled management meetings. Measurement is conducted for each relevant manager group: all the managers directly responsible for a work group in a store (online department and store managers), and managers with responsibility for groups of stores, known as “above-store managers.” Because of this, data for a manager with responsibility above a single store level may be considered when evaluating the implementation process for multiple stores under their direction, while SoC data for a store manager would only be considered in the process evaluation for the individual store under their management.

Movement through the SoC at each timepoint will be reported, as well as the absolute difference in stages, if any, from baseline to follow-up (ie, the total number of stages the work group’s managers moved through during the study period and whether movement was progressive or regressive through the SoC). This survey tool was selected as it has been previously validated as an instrument sensitive to detecting both workers’ and managers’ readiness to implement changes to address physical and psychosocial risk factors in the workplace [36,50,51]. SoC data will be collected and managed using REDCap (Research Electronic Data Capture; Vanderbilt University) electronic data capture tools hosted at La Trobe University [52,53]. REDCap is a secure, web-based software platform designed to support data capture for research studies.

A summary of outcome measures, analysis metrics, methods of aggregation, and measurement timepoints is included in Table 1.

**Table 1:** Study measures, metrics, methods of aggregation, and frequency of measurement.

Primary or secondary	Outcome measure	Analysis metric	Method of aggregation	Participants providing data	Timepoints
Primary	Physical hazard measures	Change from baseline	Mean	Individual employees from each work group	At commencement and 12 months after commencement
Primary	Psychosocial hazard measures	Change from baseline	Mean	Individual employees from each work group	At commencement and 12 months after commencement
Secondary	Change in self-rated pain and discomfort	Change from baseline	Mean	Individual employees from each work group	At commencement and 12 months after commencement
Secondary	Change in self-rated stress score	Change from baseline	Mean	Individual employees from each work group	At commencement and 12 months after commencement
Secondary	Action plan implementation	Number of actions implemented by action status or type	Count	Risk management teams from intervention stores	At the conclusion of the study
Additional	Stage of change progression	Change from baseline	Difference between initial and final values; changes between each measurement period	Store managers (allocated directly to a store or work group); above-store managers (overseeing multiple work groups)	At commencement, and every 3 months after commencement

#### Process Evaluation

Data will be collected to enable a process evaluation of the implementation of the toolkit in intervention work groups. Observations and reflections will be recorded by facilitators after each interaction with participating clusters using an online notebook and synthesized to enable evaluation of the intervention using the “Reach, Effectiveness, Adoption, Implementation, and Maintenance” framework [54]. Observation themes will include the extent and nature of engagement of participants; reflections and feedback on participants’ comments about the intervention process and their satisfaction with the process; participants’ willingness to adopt the toolkit process and identified action plans; adherence to and deviation from toolkit processes; maintenance of implementation of toolkit processes; and actions that were implemented, to the extent possible considering the duration of the study. Observations will be recorded after each face-to-face meeting with each cluster, noting which resources from the APHIRM toolkit were used and how the resources were used. Interactions with members of the RMT or store-level managers for each cluster will be noted. Observations for above-store managers will be either linked to the clusters relevant to the observation being recorded or recorded as a separate record where there is no clear link between the interaction and specific clusters. These observations will be recorded in an online notebook by facilitators and monitored and reviewed by the research team.

This data will also be used to evaluate how different clusters use the resources provided in the APHIRM toolkit and how this relates to the cluster’s SoC.

### Sample Size

Sample size calculations were based on primary outcome measures of changes in physical and psychosocial hazard severity ratings. Considerations were made to the organization’s structure when determining the number of clusters and participant numbers in each cluster. Individual stores are organized into “groups” of 10-11 stores, overseen by a group manager, justifying a 3-level cluster design.

Online department numbers were reviewed by store to determine expected cluster size. Additionally, response rates to previous surveys (not connected with the current project) were reviewed and found to be between 20% and 30%. These values were then used to calculate the sample size within each cluster to account for differences in expected recruitment across clusters. The mean cluster size (number of expected participants in a cluster) was set at 15, with a cluster variation of 0.7. If the mean cluster size deviates significantly from the estimated value of 15, this will be identified and accounted for in the statistical analysis and the interpretation of results.

Table 2 sets out the sample size and power calculations for primary and secondary outcomes evaluated in the randomized controlled trial, taking into account the 3-level cluster design at α=.05, intraclass correlation coefficient level 2=0.10; intraclass correlation coefficient level 3=0.05, mean cluster size=15; and estimated cluster variation=0.7, derived using Monte Carlo simulation. A previous study was used to estimate the effect size for exposure to physical hazards, exposure to psychosocial hazards, and self-rated pain and discomfort scores [55]. Results from Rahimi [56] were used to estimate effect sizes for stress scores.

On this basis, it was determined that 9 control and 9 intervention clusters (18 clusters in total) were required to achieve power of 0.8 or more.

**Table 2:** Sample size and power calculations.

Outcome			Mean (SD)		Effect size (*d*)		Groups (level 3), count		Stores (level 2), count		Employees (level 1), approximate n		Power (95% CI)
**Primary outcomes**													
	Exposure to physical hazards	3.7 (0.6)		0.5		3		18		270		0.870 (0.816-0.910)	
	Exposure to psychosocial hazards	2.4 (0.6)		0.5		3		18		270		0.870 (0.816-0.910)	
**Secondary outcomes**													
	Pain/discomfort	14.9 (8.3)		4		3		18		270		0.780 (0.753-0.805)	
	Stress	4.02 (2.56)		0.5		3		18		270		0.742 (0.714-0.768)	
	Burnout	4.76 (2.54)		0.5		3		18		270		0.734 (0.706-0.760)	
	Cognitive stress	3.33 (2.57)		0.5		3		18		270		0.745 (0.717-0.771)	

### Randomization

**Figure 2:**
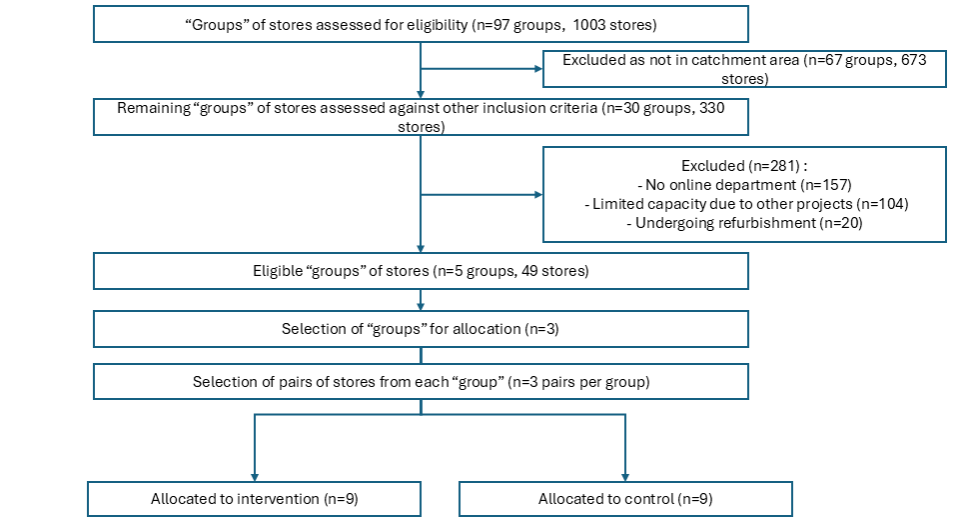
Participant flow for recruitment to the study.

The exclusion criteria were applied to a complete list of “groups” of stores in the organization prior to randomization. Groups of stores were excluded where the number of eligible stores in the group fell below threshold. Figure 2 illustrates participant flow by cluster for recruitment to the study.

A list of eligible groups of 10-11 stores was provided to an independent researcher to perform randomization using a random number generator based on the stores’ numeric identifier and assign them to the control or intervention arm of the study.

A total of 3 groups of stores that met the eligibility criteria were randomly selected first, and then 6 stores were randomly selected from each of those groups. Clusters (stores) were randomly allocated to either the control or intervention arm. Pairs were not matched. Consent was not obtained at the time of recruitment and randomization of stores, as consent procedures will be followed when recruiting individual participants during data collection procedures, such as administration of the APHIRM toolkit survey or the SoC questionnaire.

### Blinding

Blinding of cluster allocations was not possible, as each cluster communicates with the other clusters in the study on a regular basis and would thus be able to determine their allocation in the study.

Raw data from the toolkit will be reviewed by a statistician on the research team who is blinded to cluster allocation.

### Statistical Methods

Baseline characteristics, including baseline values of the primary outcomes, store size (number of employees), and available aggregate demographic measures, will be summarized at the store level and presented descriptively by treatment arm (mean, SD, median, range, or proportion, as appropriate). Data for each cluster will be analyzed to identify potential outliers. Data will be cleaned and examined for normal distribution prior to analysis. The primary outcome measures (physical and psychosocial hazard severity ratings) will be analyzed by variance-weighted cluster-level ANCOVA using posttreatment store means regressed on treatment assignment and baseline store mean, with group fixed effects. Weights will be set to the inverse of the estimated theoretical variance of the cluster means to account for potential unequal cluster sizes. Because there are only 18 clusters (9 per arm) and 3 higher-level strata, estimation of higher-level variance components is unstable, and asymptotic cluster-robust SEs perform poorly. However, analyses using generalized estimating equations will be presented as sensitivity checks, with full disclosure of small-sample limitations.

Data on action plan implementation will be analyzed as categorical data. The count of actions by each status category (eg, “completed,” “in progress,” or “not started”) and categorization as “higher-order” or “lower-order” will be performed for each cluster and compared against the other outcome measures for that cluster. Data on SoC progression will be analyzed as ordinal data. The median and mode response for the cluster will be determined as the SoC for that cluster at each measurement point. SoC data for store managers and above-store managers will be analyzed separately for each cluster. Progression through the SoC will be evaluated by assessing the number of SoC each group progresses through at each timepoint and overall, from commencement to the conclusion of the study.

### Missing Data

The APHIRM toolkit survey requires completion of each item before the next one is presented, so a missing data strategy is not required. The SoC questionnaire is also managed in the same way. Consequently, a plan for handling missing data with respect to these data is not required.

Facilitators submit a completed worksheet at the end of each session completed with each cluster. This is used to evaluate adherence to protocol and to investigate and document any deviations from the protocol. These will be documented in the records maintained for each cluster, which are used to complete the process evaluation for this study.

## Results

Funding for the study was approved in June 2025. Ethics approval for the study was granted in July 2025. Recruitment and randomization commenced in August 2025. This included identification of eligible stores and randomization of the eligible stores to the control or intervention groups until the required sample size was achieved. Data collection commenced in September 2025. These timings resulted from a necessary operational requirement to avoid key study activities coinciding with busy trading periods, such as Christmas and Easter, without delaying the commencement of the study. As of January 2026, a total of 18 stores were recruited to the study, with 332 individuals responding to the APHIRM toolkit survey. Response rates to the APHIRM toolkit survey for each cluster range between 28% and 85%, with an average response rate of 47% of all eligible employees. Data have not been viewed by the research team. The intervention is expected to conclude in September 2026, and results are anticipated to be published in early 2028.

## Discussion

### Generalizability

WMSDs and MHPs have a significant impact on Australian workers and workplaces, including the retail trade sector [57]. Recommendations from research on WMSD and MHP prevention advocate for a participatory approach, focusing on the whole job rather than discrete tasks, and taking into account both physical and psychosocial hazards [18,58,59]. Previous research has highlighted the evidence-to-practice gap in prevention practices, including those used by safety professionals in the retail sector [5]. This study aims to translate research evidence into practice and address WMSD and MHP hazards through implementation of the APHIRM toolkit. This study seeks to understand what support managers and decision makers require to facilitate effective implementation of these practices in their area of control. This is an important evidence gap in understanding the manager’s role in successful workplace interventions [36,41,60]. It is anticipated that these findings could be used to recommend future improvements to the design and content of supporting resources in the APHIRM toolkit.

The retail organization hosting this study is one of Australia’s largest employers, with more than 1100 locations across the country. This provides an opportunity to examine the study’s research questions at a level of organizational scale and complexity that is often not possible in Australia. The design also provides an opportunity to understand how to support large, multisite employers to bridge the evidence-to-practice gap with respect to WMSD and MHP risk management. This is important for employers, who, due to organizational size, complexity, and geographic spread, must rely on nonexperts to implement and maintain risk management practices with respect to WMSD and MHP prevention. The design of the study endeavors to replicate real-world conditions of toolkit implementation as closely as possible so that findings can be generalized to other work groups in the organization as much as possible. However, the availability of staff in the organization to act as facilitators, and their location—proximal to state capital cities—may present a limitation to the generalizability of findings to regional and remote work group locations.

As part of identifying recommendations to improve these practices, this study provides an opportunity to understand how managers at different levels of a large organization, and in different physical locations, progress through SoC during implementation of the APHIRM toolkit. The study also presents an opportunity to understand how managers’ progression through SoC relates to outcomes from toolkit implementation. It is anticipated that this study could also inform how the APHIRM toolkit could be best used, and possibly adapted, for use in large multisite organizations, in ways that support managers of work groups at different SoC. The intervention strategy aims to replicate how the toolkit may be implemented within a large organization, which serves to build the capacity of individual stores, their leadership teams, and their supporting safety professionals to more effectively manage the risks of WMSDs and MHPs. It is anticipated that the findings of the study could be applied to other large, multisite organizations, particularly in the Australian retail sector.

### Limitations

Limitations on facilitators’ ability to travel and other time constraints prevent the recruitment of work groups located in regional or remote locations, which typically encounter different operational challenges compared with those located closer to a capital city. This should be considered when translating the results of this study to work groups in these geographies.

All work groups in this study are recruited from the same organization. As such, changes at a whole-of-systems level could possibly impact control and intervention stores alike, thereby influencing the results of this study. This presents a challenge when attempting to attribute changes in outcome measures to any one management level or team within the organization. However, it is anticipated that RMTs in stores will identify a combination of actions to address identified hazards and risks that are specific to their individual store, as well as actions that are applicable at the level of the system of work. Consequently, RMTs will require assistance from personnel above the individual store level to address system-related actions. The implementation of actions at the store level is expected to assist in differentiating intervention stores from control stores when measuring hazard severity at follow-up. The impacts of these systems level changes are expected to be felt by control and intervention stores equally, as it is anticipated these changes will be implemented at all stores. However, it is also highly likely that the majority of systems-level changes will not be implemented prior to the conclusion of the study. It is expected that the majority of any observed changes will be attributable to store-level changes, which can be implemented in a shorter amount of time.

It is also possible that the act of engaging RMTs from the intervention stores in the development of above-store actions will have an impact on hazard severity scores at follow-up, greater than the impact of the implementation of actual changes to the system of work.

## Appendix

Multimedia Appendix 1 Stage of change questionnaire for managers.

## Abbreviations

APHIRM: A Participatory Hazard Identification and Risk Management
MHP: mental health problem
MSD: musculoskeletal disorder
REDCap: Research Electronic Data Capture
RMT: risk management team
SoC: Stages of Change
WMSD: work-related musculoskeletal disorder
