# Supplementary material for: Implementation of a Participatory Ergonomics Intervention to Reduce Musculoskeletal and Stress-Related Mental Health Risks in Australian Retail Workers: Protocol for a Randomized Controlled Trial
Source: JMIR Res Protoc. 2026 Jan 29;15:e84864. doi: 10.2196/84864 (PMC12902761; doi:10.2196/84864)
Supplement: Multimedia Appendix 1 [file resprot_v15i1e84864_app1.docx]

# Stage of Change Questionnaire for Managers

| Question | Response options and scoring algorithm |
| --- | --- |
| Are you concerned about manual handling (MSD) injuries in your store/ group? | Yes - move to next stage  No - in precontemplation stage |
| Are you thinking about making changes to reduce MSD injuries in the next 6 months? | Yes - move to next stage  No - in contemplation stage |
| Do you have a clear idea of what action you want to take? | Yes - move to next stage  No - in contemplation stage |
| Are you planning to make these changes in the next month or two? | Yes - move to next stage  No - in preparation stage |
| Have changes already been made? | Yes - move to next stage  No - in action stage |
| If so, describe the changes: |  |
| If you made changes in the last 6 months, do you have any further actions planned? | Yes - move to next question  No - in maintenance stage |
| If yes, describe the changes: |  |
| Which hazards do you think contribute to MSD injuries? Choose all that apply from the following list: | Moving heavy items  Doing repetitive work  High workloads  Low decision making about when you do certain tasks and/or how they are done  Poor relationships with managers  Working in awkward postures  Being older |
| I am confident at identifying changes to make when a team member reports a hazard that could cause an MSD (e.g., not enough room for totes in the Online room, or not receiving feedback on performance) | 1 = not at all  7 = very confident |
| I feel confident asking my team about what changes they think we could make to manage MSD hazards in our store. | 1 = not at all  7 = very confident |
| I think my manager and their managers believe it is important to focus on identifying and managing hazards. | 1 = not at all important  7 = very important |
| I have the tools and resources I need to identify, assess, and manage hazards that could cause an MSD. | 1 = not at all  7 = I have everything I need |
| I recognize team members who identify hazards that could cause an MSD and/or make suggestions about how to manage them. | 1 = not at all  7 = almost always |
